# Supplementary material for: The HORMA-domain protein ASY1 recruits the plant-specific cyclin SDS to promote DMC1-mediated meiotic recombination
Source: Plant Cell. 2026 Jun 11;38(8):koag176. doi: 10.1093/plcell/koag176 (PMC13440570; doi:10.1093/plcell/koag176)
Supplement: Supplementary file 1 [file koag176_supplementary_data.zip › cai et al_Supplementary information.pdf]

Supplementary figures and legends

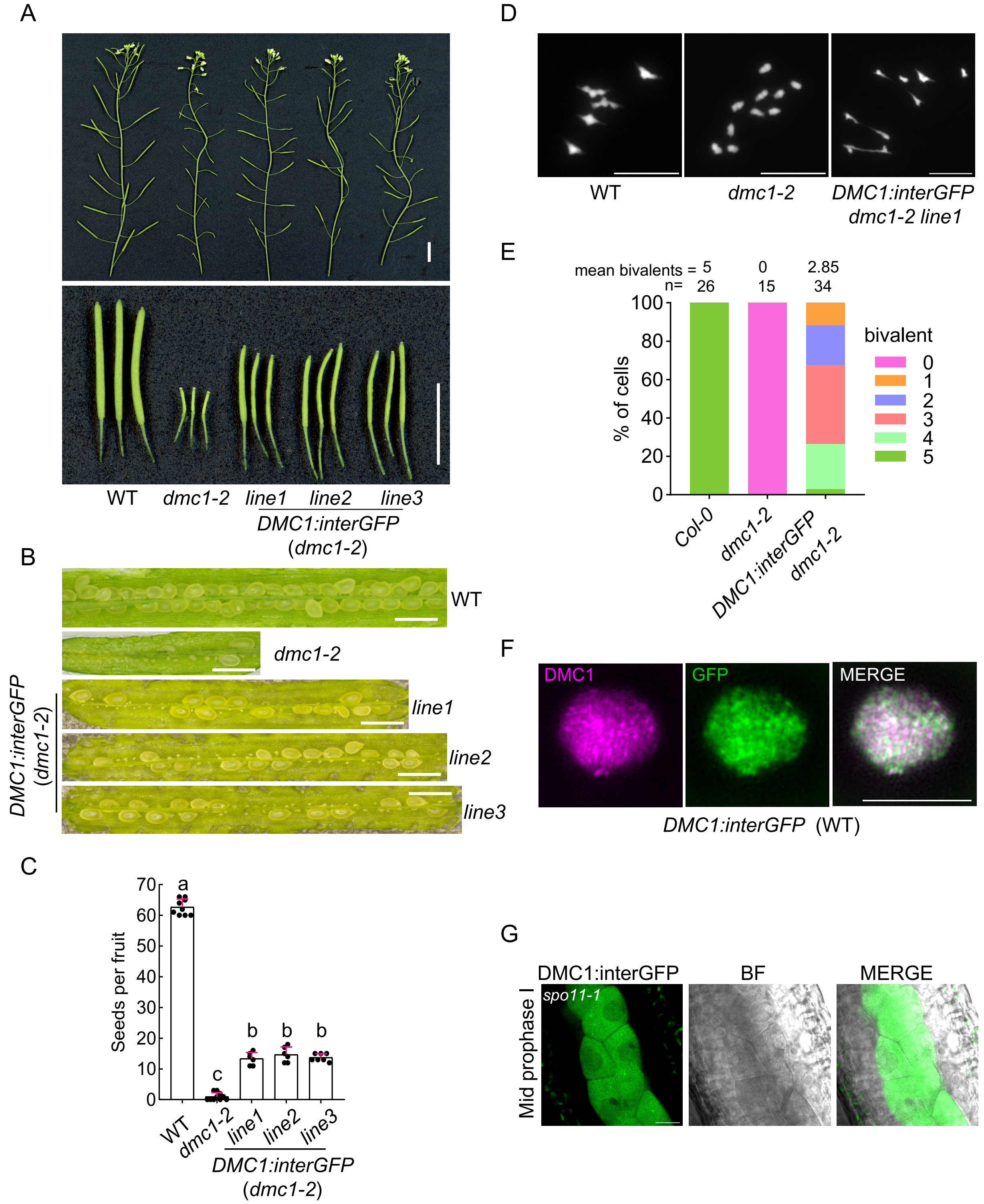

Supplementary Figure 1. The *DMC1:GFP* reporter is partially functional (Supports Figure 1). (A) The main branches (upper panel) and siliques (low panel) of WT, *dmc1-2* and *DMC1:interGFP* (*dmc1-2*) three independent lines. Bars=1 cm. (B) The seed sets of WT, *dmc1-2* and *DMC1:interGFP* (*dmc1-2*) three independent lines. (C) Statistical analysis of seed sets according to (B). Different small letters indicate significant difference (Tukey's

multiple comparison test, $P<0.05$). (D) Chromosome spread analysis of male meiocytes from wildtype, *dmc1-2*, and *DMC1:interGFP* (*dmc1-2*) line1. Bars=10 μm. (E) Quantification of chromosome configurations at metaphase I in wildtype (n=26 cells), *dmc1-2* (n=15 cells), and *DMC1:interGFP* (*dmc1-2*) (n=34 cells) line1. (F) Co-immunostaining using DMC1 and GFP antibodies in wildtype plants harboring *DMC1:interGFP*. Bar=10 μm. (G) Live-cell confocal images showing the localization pattern of DMC1:interGFP in male meiocytes of *spo11-1* mutants. Scale bars=10 μm.

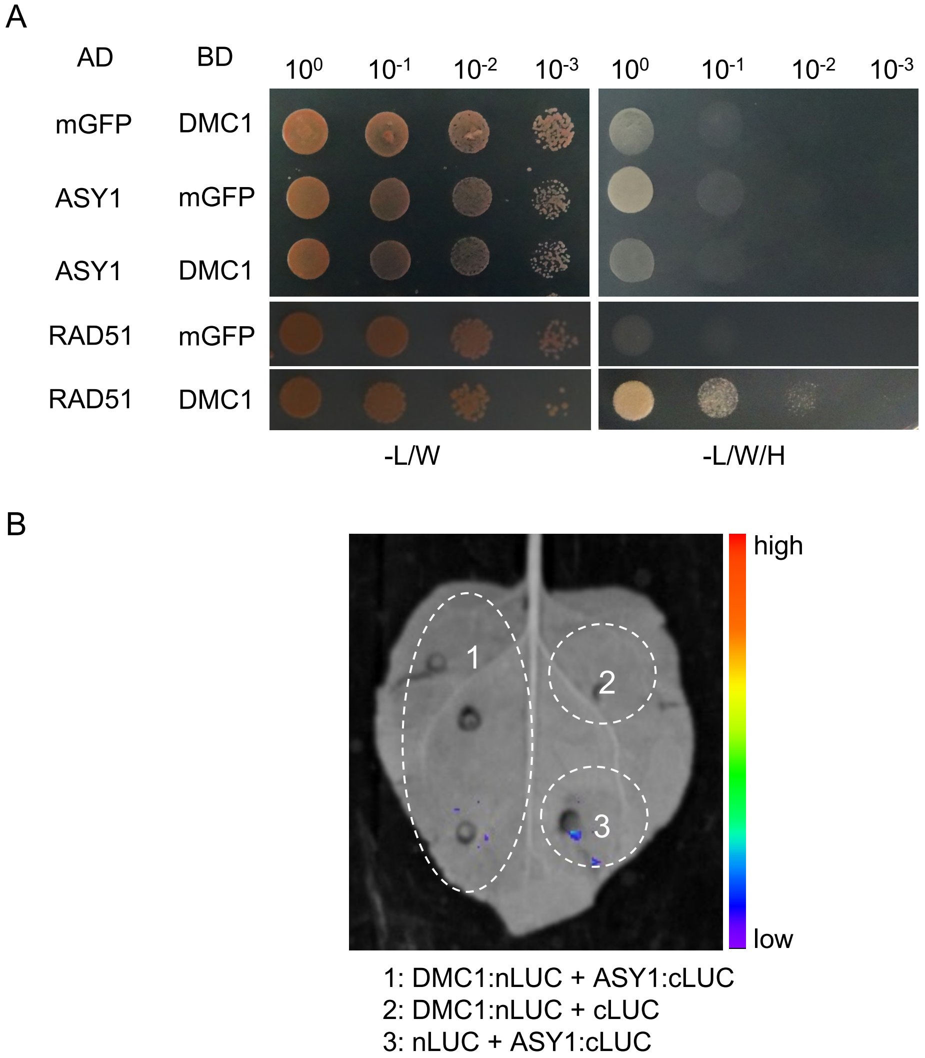

Supplementary Figure 2. ASY1 has no direct physical interaction with DMC1 (Supports Figure 1 and 5). (A) Yeast two-hybrid assay for testing the interaction between ASY1 and DMC1. Monomeric GFP (mGFP) fused with AD (activating domain) and BD (binding domain) were used as controls. The combination of RAD51 and DMC1 was shown as positive control. (B) Split-luciferase complementation assay for the interaction of ASY1 with DMC1 using *N. Benthamiana* leaves. ASY1 and DMC1 were fused with the C- or N-terminal parts of the luciferase, respectively. The unfused forms of nLUC or cLUC were used as negative controls.

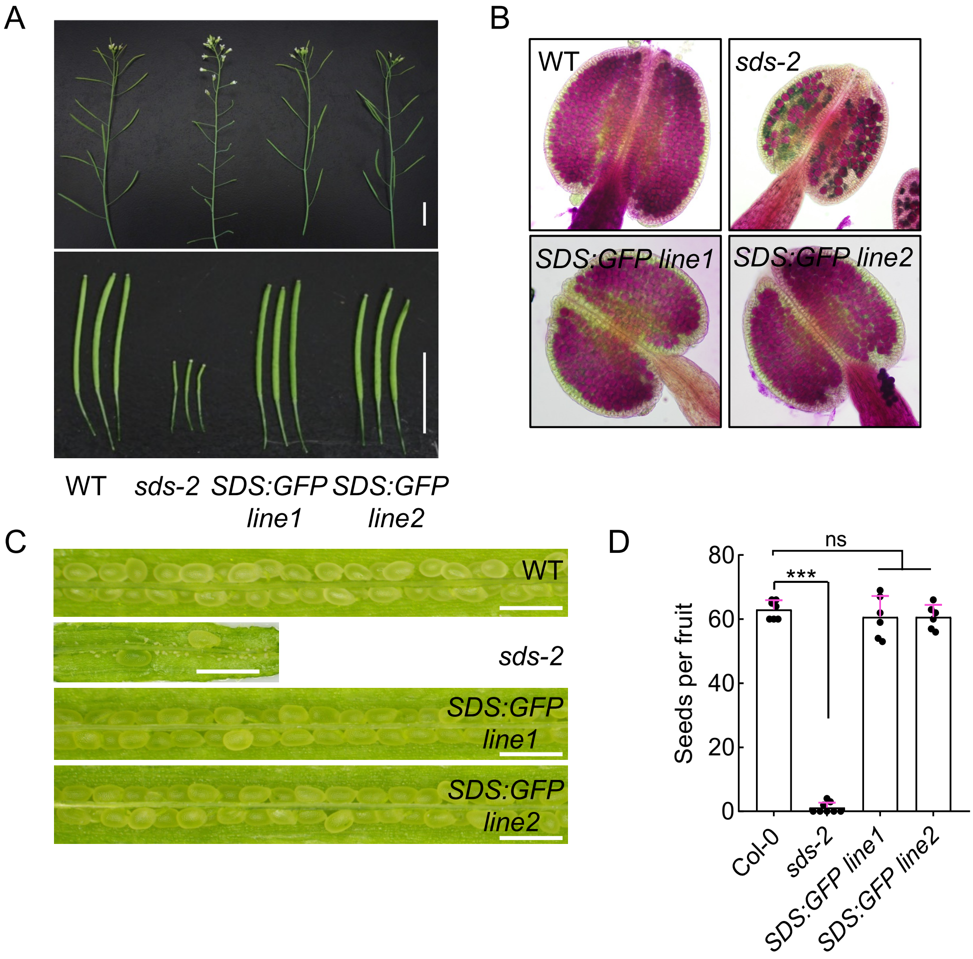

Supplementary Figure 3. The SDS:GFP reporter is functional (Supports Figure 2). (A) The main branches (upper panel) and siliques (low panel) of WT, *sds-2*, and two lines of *SDS:GFP* (*sds-2*) plants. Bars: 1 cm. (B) Pollen staining of WT, *sds-2*, and two lines of *SDS:GFP* (*sds-2*) plants. (C) The seed sets of WT, *sds-2*, and two lines of *SDS:GFP* (*sds-2*) plants. (D) Statistical analysis of seed sets according to (C). Error bars indicate mean ± SD. Tukey's multiple comparaison test, *** $P < 0.001$.

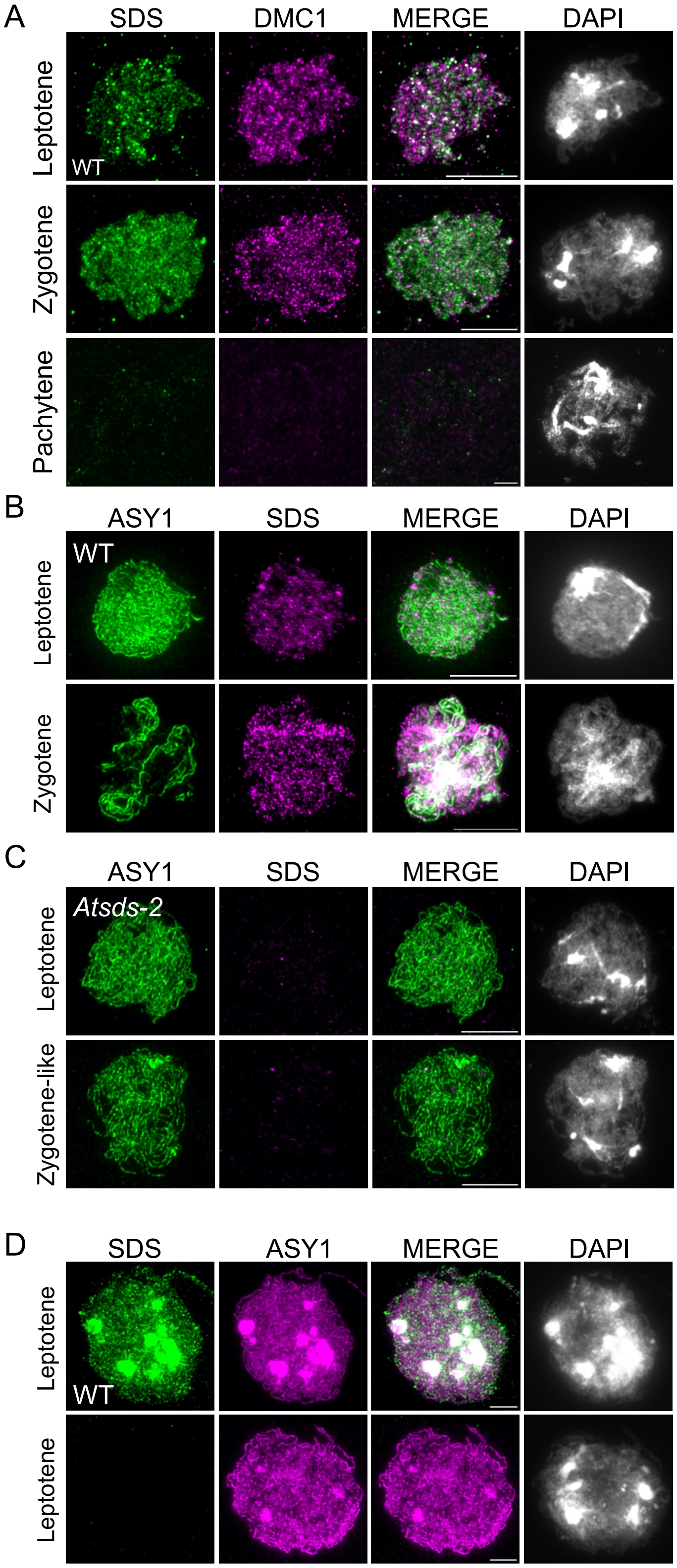

Supplementary Figure 4. The specificity validation of SDS antibody (Supports Figure 2). (A) Co-immunostaining of SDS and DMC1 in male meiocytes of the wildtype Arabidopsis during prophase I. (B) Co-immunostaining of ASY1 and SDS in male meiocytes of the wildtype Arabidopsis during prophase I. (C) Co-immunostaining of ASY1 and SDS in male meiocytes of Arabidopsis *sds* mutants during prophase I. Scale bars=10

μm. (D) Co-immunostaining of ASY1 and SDS in wildtype *B. napus* male meiocytes at leptotene. Note that in the lower panel, the BnaSDS antibody was pre-incubated with the recombinant antigen proteins. Scale bars=5 μm.

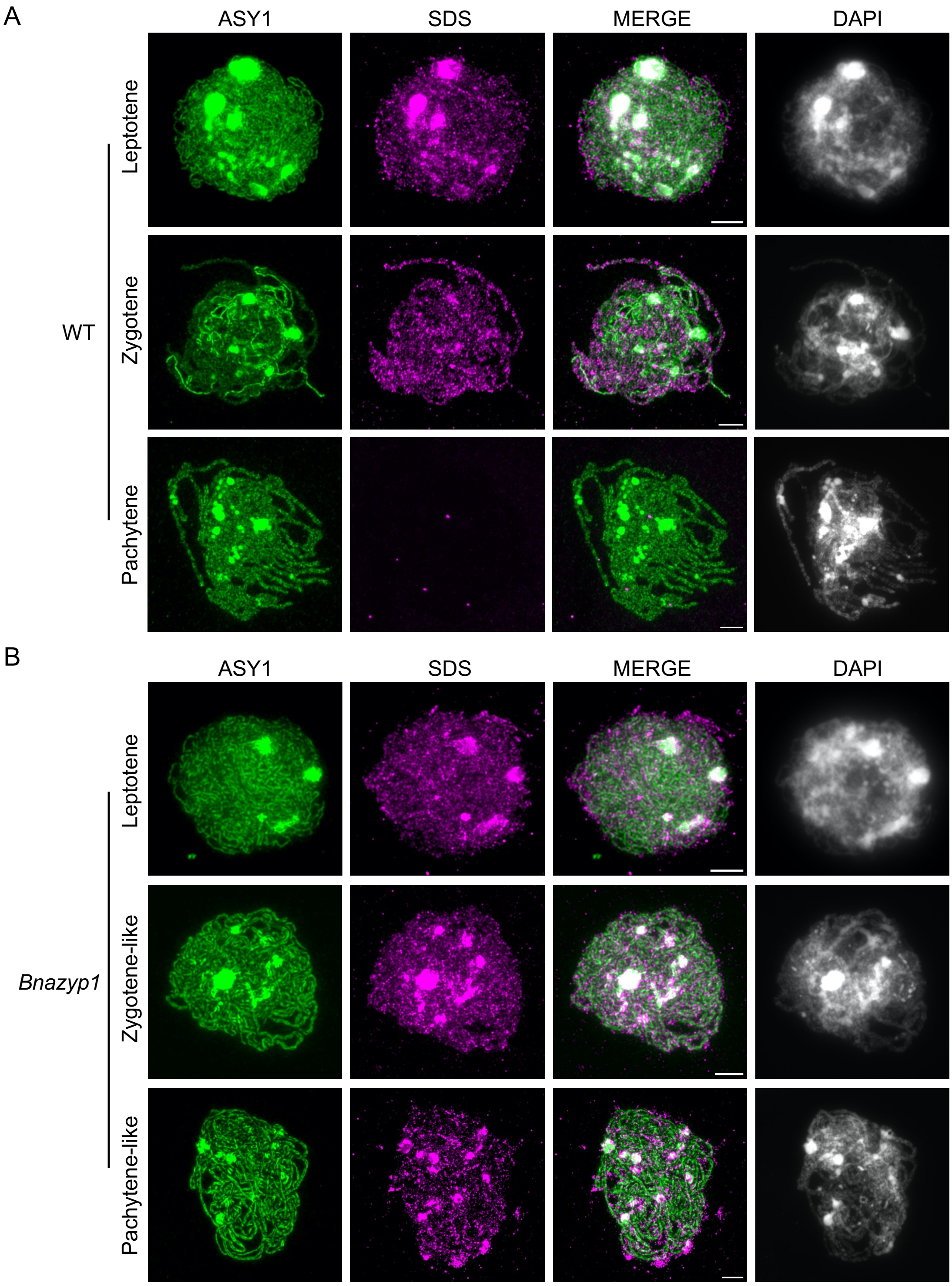

Supplementary Figure 5. SDS exhibits prolonged stay on meiotic chromosomes in *Brassica napus zyp1* mutants (Supports Figure 2 and 6). (A) Co-immunolocalization of SDS and ASY1 in male meiocytes of the wildtype *Brassica napus* at different stages of prophase I. (B) Co-immunolocalization of SDS and ASY1 in male meiocytes of the *Brassica napus zyp1* mutants at different stages of prophase I. Scale bars=5 μm.

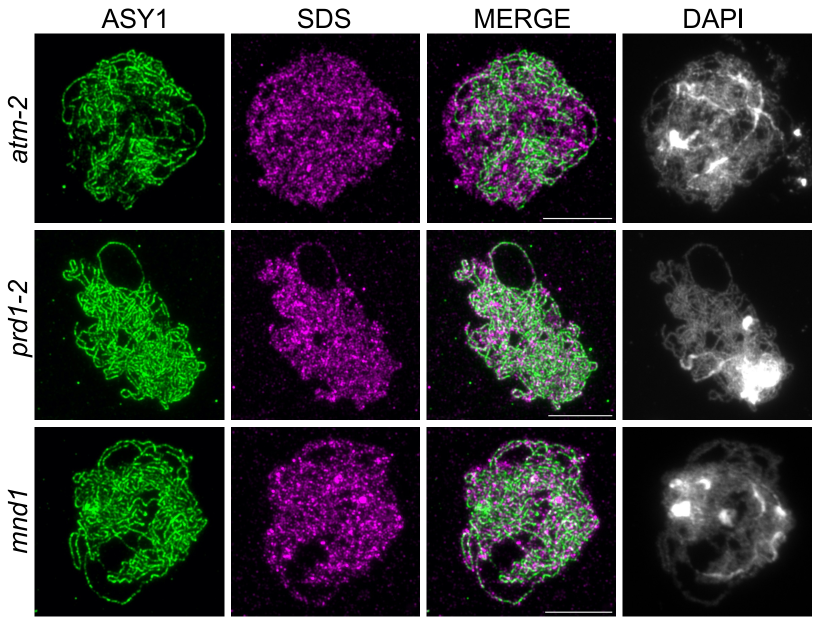

Supplementary Figure 6. SDS localization on chromosomes is not affected in the absence of ATM, PRD1, or MND1 (Supports Figure 4).. Co-immunolocalization of ASY1 and SDS in *atm-2*, *prd1-2*, and *mnd1* mutants. Scale bars=10 µm.

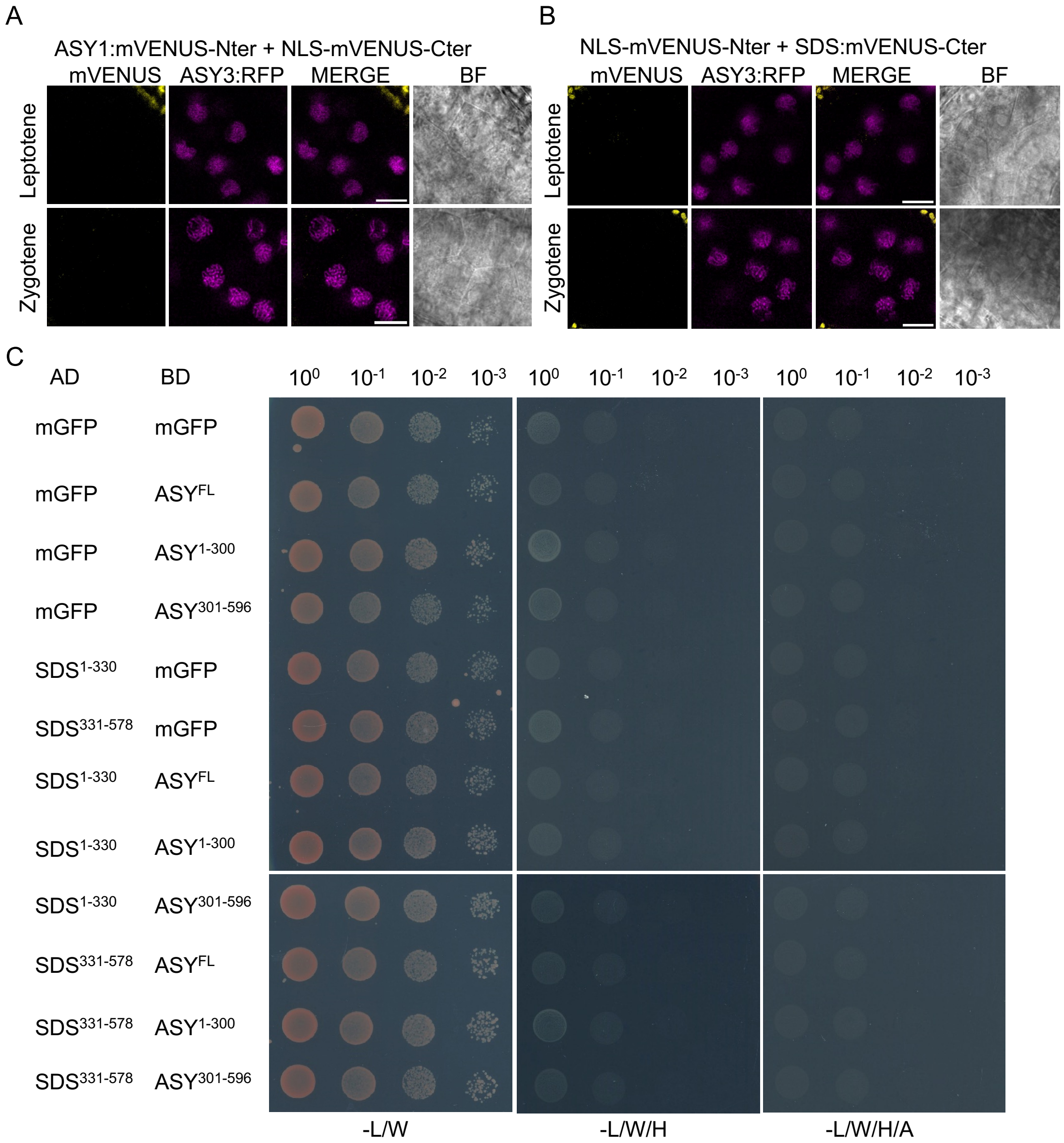

Supplemental figure 7. Experimental controls for *in planta* BiFC and yeast two-hybrid assay between ASY1 and SDS (Supports Figure 5). (A) The BiFC assay for the combination of ASY1:mVENUS^{Nter} with NLS-mVENUS^{Cter}. Scale bars=10 μm. (B) The BiFC assay for the combination of SDS:mVENUS^{Cter} with NLS-mVENUS^{Nter}. All images were captured from Arabidopsis male meiocytes of wildtype during prophase I. Scale bars=10 μm. (C) Yeast two-hybrid assay for testing the interaction between truncated SDS and full-length ASY1 or ASY1 truncations.

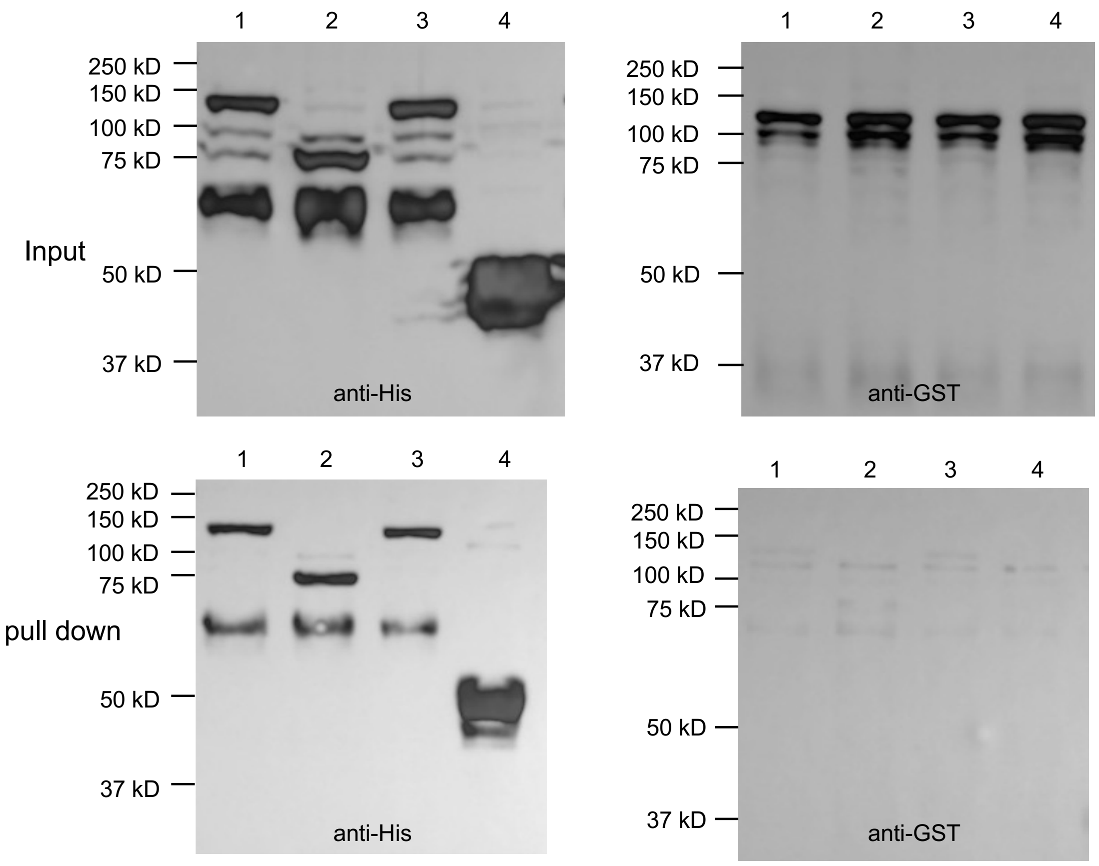

1. GST-SDS-pGGWA + HisMBP-ASY1^{FL}-pHMGWK

2. GST-SDS-pGGWA + HisMBP-ASY1$^{1\text{-}300}$-pHMGWK

3. GST-SDS-pGGWA + HisMBP-ASY1$^{1\text{-}570}$-pHMGWK

4. GST-SDS-pGGWA + HisMBP-pHMGWK

Supplemental figure 8. Pull-down assays between ASY1 and SDS (Supports Figure 5). Ni^{2+} pull-down assays of GST-SDS with HisMBP-ASY1^{FL}, HisMBP-ASY1$^{1\text{-}570}$, or HisMBP-ASY1$^{1\text{-}300}$. HisMBP was used as negative control. The input and pull-down fractions were analyzed by immunoblotting with the anti-GST and anti-His antibodies.

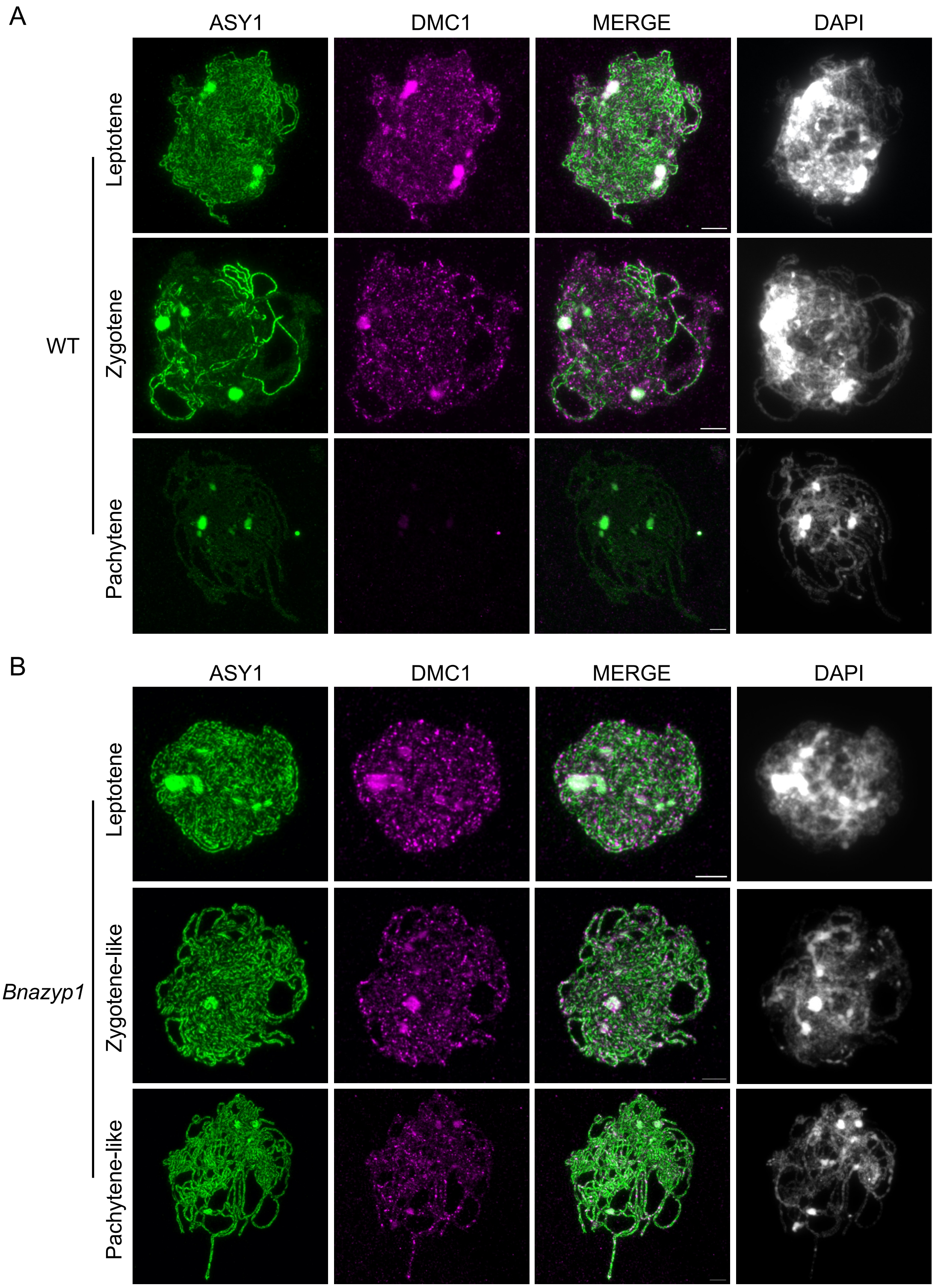

Supplementary Figure 9. DMC1 exhibits prolonged stay on meiotic chromosomes in *Brassica napus zyp1* mutants (Supports Figure 6). (A) Co-immunolocalization of DMC1 and ASY1 in male meiocytes of the wildtype *Brassica napus* at different stages of prophase I. (B) Co-immunolocalization of DMC1 and ASY1 in male meiocytes of the *Brassica napus zyp1* mutants at different stages of prophase I. Scale bars=5 μm.

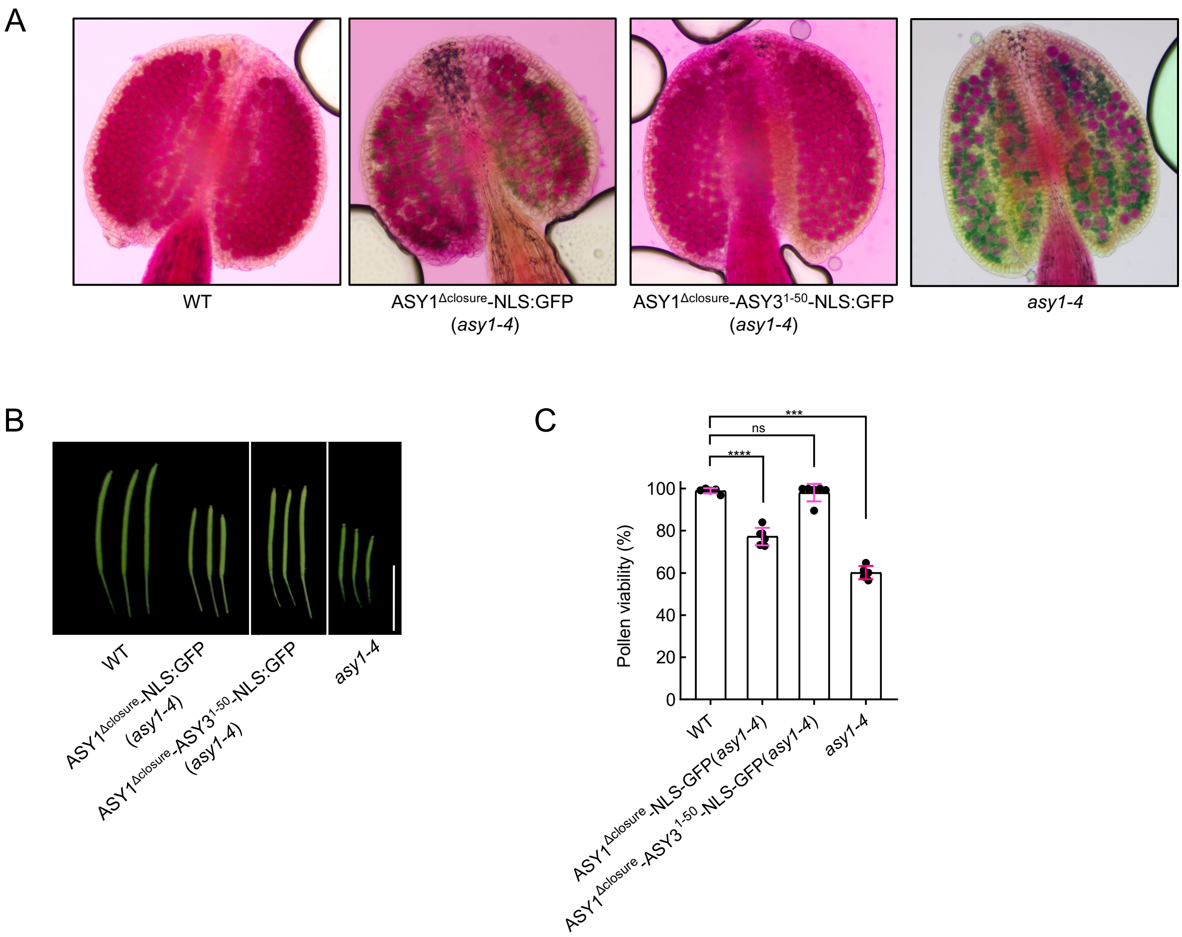

Supplementary Figure 10. Fertility analysis of *asy1* mutants haboring different ASY1 variants (Supports Figure 7). (A) Pollen staining of WT, *ASY1*$^{\Delta closure}$*-NLS:GFP* (*asy1-4), ASY1*$^{\Delta closure}$*-ASY3*$^{1\text{-}50}$*-NLS:GFP* (*asy1-4*) and *asy1-4* plants. (B) Siliques of WT, *ASY1*$^{\Delta closure}$*-NLS:GFP* (*asy1-4), ASY1*$^{\Delta closure}$*-ASY3*$^{1\text{-}50}$*-NLS:GFP* (*asy1-4*) and *asy1-4* plants. Bars: 1 cm. (C) Statistical analysis of pollen viability according to (A). *** $P<0.001$ (Tukey's multiple comparison test). Error bars indicate mean ± SD.

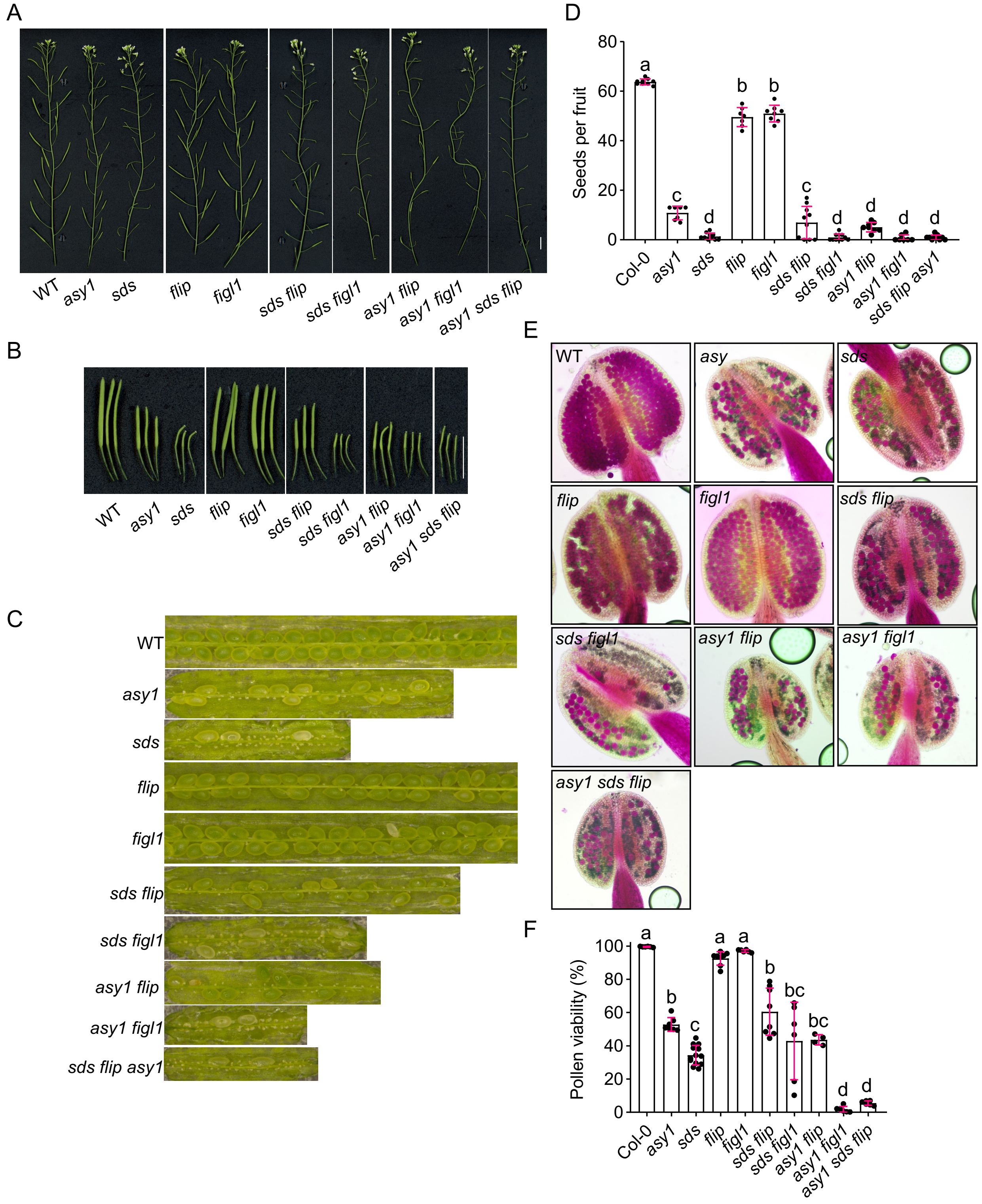

Supplementary Figure 11. Phenotypic analysis of wildtype, *asy1*, *sds*, *flip*, *figl1*, *sds flip*, *sds figl1*, *asy1 flip*, *asy1 figl1*, and *asy1 sds flip* (Supports Figure 8). (A-B) The main branches (A) and siliques (B) of WT, *asy1*, *sds*, *flip*, *figl1*, *sds flip*, *sds figl1*, *asy1 flip*, *asy1 figl1*, and *asy1 sds flip* plants. Bars: 1 cm. (C) The seed sets of WT, *asy1*, *sds*, *flip*, *figl1*, *sds flip*, *sds figl1*, *asy1 flip*, *asy1 figl1*, and *asy1 sds flip* plants. (D) Quantification of seed sets according to (C). (E) Pollen staining with intact anthers from WT, *asy1*, *sds*, *flip*,

figl1, *sds flip*, *sds figl1*, *asy1 flip*, *asy1 figl1*, and *asy1 sds flip* plants. (F) Quantification of pollen viability according to (E). Error bars in (D) and (F) indicate mean ± SD and different small letters indicate significant difference (Tukey's multiple comparison test, P<0.05).
